# Evaluating a selective prevention program for substance use and comorbid behavioral problems in adolescents with mild to borderline intellectual disabilities: Study protocol of a randomized controlled trial

**DOI:** 10.1186/s12888-015-0563-1

**Published:** 2015-07-22

**Authors:** Esmée P. Schijven, Rutger C.M.E. Engels, Marloes Kleinjan, Evelien A.P. Poelen

**Affiliations:** Research and Development, Pluryn, P.O. Box 53, , 6500 AB Nijmegen, The Netherlands; Behavioral Science Institute, Radboud University Nijmegen, P.O. Box 9104, , 6500 HE Nijmegen, The Netherlands; Trimbos Institute, Netherlands Institute of Mental Health and Addiction, Utrecht, The Netherlands

**Keywords:** Substance use, Intellectual disabilities, Intervention, Adolescents, Study protocol

## Abstract

**Background:**

Substance use and abuse is a growing problem among adolescents with mild to borderline intellectual disabilities (ID). Substance use patterns in general population are similar to patterns among non-disabled peers, but substance use has more negative consequences for adolescents with mild to borderline ID, and they are at an increased risk for developing a substance use disorder. Nevertheless, effective and evidence based prevention programs for this groups are lacking. The study described in this protocol tested the effectiveness of a selective intervention aimed at reducing substance use in adolescents with mild to borderline ID and behavioral problems. In the intervention, participants acquire competences to deal with their high-risk personality traits.

**Methods:**

A randomized controlled trial will be conducted among 14–21-year old adolescents with mild to borderline ID and behavioral problems admitted to treatment facilities in the Netherlands. Inclusion criteria are previous substance use and personality risk for substance use. Participants will be individually randomized to the intervention (n = 70) or control (n = 70) groups. The intervention group will be exposed to six individual sessions and five group sessions carried out by two qualified trainers over six-week period. Primary outcomes will be the percentage reduction in substance use (for alcohol: percentage decrease of binge drinking, weekly use and problematic use, for cannabis: the percentage decrease of lifetime cannabis use and weekly use and for hard drug: the percentage decrease of lifetime use). Secondary outcomes will be motives for substance use, intention to use, and internalizing and externalizing behavioral problems. All outcome measures will be assessed after two, six, and twelve months after the intervention.

**Discussion:**

This study protocol describes the design of an effectiveness study of a selective prevention program for substance use in adolescents with mild to borderline ID and behavioral problems. We expect a significant reduction in alcohol, cannabis and hard drug use among adolescents in the intervention group compared with the control group.

**Trial registration:**

This trial is registered in the Dutch Trial Register (Cochrane Collaboration) as NTR5037 registered at 15 April 2015.

## Background

Substance use and abuse is a problem among adolescents and young adults with mild to borderline intellectual disabilities (ID), and both scientists as practitioners report increasingly number of concerns about this subject [1–4]. The results of an explorative study in the Netherlands showed that 75 %-85 % of adolescents with mild to borderline ID and severe behavioral problems who are admitted to treatment facilities show lifetime alcohol use or use of alcohol on a regular basis. Moreover, 25 %-50 % of the described target group uses drugs (in particular cannabis) occasionally or regularly. For most cannabis users, cannabis is a part of their daily habit [5, 6]. Although patterns of cannabis use are quite similar to substance use patterns among non-disabled peers, this is of great concern because substance use and misuse have more negative consequences [1, 5, 7] for adolescents with mild to borderline ID. Substance use by these adolescents causes various problems, including social, mental and behavioral problems, criminal activities, and financial problems [4]. Adolescents are also at a higher risk for developing a substance use disorder [6, 8, 9] compared to their nondisabled peers. To limit these negative consequences, the development of prevention programs that are adjusted to the needs of adolescents with mild to borderline ID is necessary. However, currently well-fitting and evidence-based prevention programs for adolescents with mild to borderline ID and substance use are lacking [10–12].

### Take it personal!

‘Take it personal!’ is a selective prevention program aimed at reducing substance use in adolescents with mild to borderline ID [13]. The program is specifically developed for adolescents with mild to borderline ID who receive treatment for additional behavioral problems. It is a selective intervention targeting adolescent who initiated substance use and who have a personality risk for substances. ‘Take it personal!’ offers adolescents competences to deal with their personality traits and associated motives for excessive substance use. The program is based on an existing program for non-disabled peers that have been proven to be effective [14–17] and is based on the theory that personality is an important construct for understanding adolescents’ substance use and misuse [14]. Four personality profiles are identified to be associated with substance use namely, Sensation Seeking (SS), Impulsivity (IMP), Anxiety Sensitivity (AS), and Negative Thinking (NT) [14]. Each personality profile is associated with unique patterns of substance use, maladaptive motives for substance use, and comorbid psychopathology [18–20]. Sensation seekers are more likely to be heavy drinkers and have greater risk for adverse drinking consequences [21, 22]. Impulsivity is associated with increased risk for early onset of alcohol and drug problems [23]. The lack of ability to delay behavioural response in impulsive individuals [24] is a risk factor for abuse of drugs due to a self-regulation deficit [25]. Highly anxious sensitive persons showed increased levels of drinking [26, 27] are more responsive to the anxiety-reducing effect of alcohol, are more likely to use alcohol to cope with negative feelings [28], and have a higher incidence of problem drinking symptoms [29]. They often cope with their negative feelings by using a combination of withdrawal (from social situations), dependence (on others to make them feel better), or use of alcohol and/or drugs. Persons with high levels of hopelessness usually use alcohol and/or drugs to cope with negative feelings [14, 28, 30, 31]. The intervention offers adolescents competences to deal with their personality traits and associated motives for substance use. Previous studies on interventions based on these personality profiles have demonstrated that this intervention is effective for adolescents with normal intelligence [14–17]. These personality profiles have not been applied in interventions targeting adolescents with mild and borderline ID, while they might be particularly relevant for this high-risk target group, as personality related substance use and psychopathology is highly prevalent in this population.

The intervention is based on the theoretical principles of motivational interviewing (MI) and cognitive behavioral therapy (CBT), the techniques that have been proven to be effective by decreasing alcohol and drug use among non-disabled adolescents [32–35]. Scientific evidence also shows that these techniques are effective for people with mild to borderline ID [36–38].

The intervention for adolescents with mild to borderline ID was developed according to the guidelines for effective interventions for people with mild ID [39]. We used simple information; offered concrete exercises and games; used more visual materials (like pictures), short sessions, and more sessions for repetition. Furthermore, we used techniques of psychomotor therapy. Psychomotor therapy is a common method used by adolescents with ID, and practitioners have good experiences in daily practice. This intervention will fill a gap in existing prevention programs for adolescents with mild to borderline ID.

### Aims and hypotheses

The main aim of this study is to test the effectiveness of the intervention ‘Take it personal!’ in decreasing substance use among adolescents (age 14 – 21 years old) with mild to borderline ID and behavioral problems who are admitted to treatment facilities in the Netherlands. The effectiveness of the intervention is being assessed by conducting a randomized controlled trail (RCT) with two conditions (treatment and control group). Follow-up assessments will be carried out at two, six, and twelve months after the start of the intervention.

The main hypothesis is that the intervention will reduce alcohol, cannabis, and hard drug use among individuals in the intervention group compared to those in the no-intervention control group. We also expect a decrease in the intention to use alcohol, cannabis and/or hard drugs in the future, a change in motives for alcohol and/or drug use after two, six, and twelfth moths in the intervention group compared with the control group. In addition, the effect of the program on internalizing and externalizing behavioral problems will be tested after two, six and twelve months after start of the intervention. We hypothesized that the intervention would decrease internalizing and externalizing behavioral problems in the intervention group compared with the control group.

## Methods/Design

### Study design

**Fig. 1:**
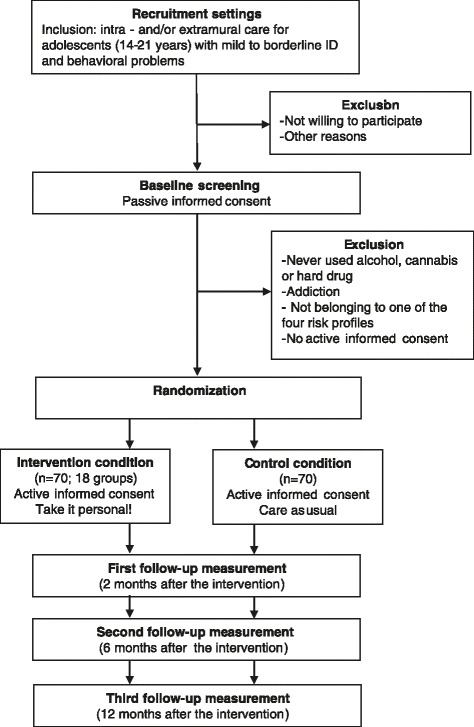
Study design

The effectiveness of the intervention will be tested in an RCT with two arms, an intervention group and a control group (see Fig. 1). The intervention effects will be tested at two, six, and twelfth months after the start of the intervention. Participants will be 140 adolescents with mild to borderline ID and behavioral problems receiving treatment in treatment facilities. The adolescents will be randomly assigned to the intervention condition (Take it personal!; n = 70) or the control condition (care as usual; n = 70). Recruitment, inclusion, and randomization of the participants will start in the beginning of 2015. The program will start in the spring of 2015 and will continue until the end of 2015. This trial is registered at the Dutch trial register (trial registration: Dutch Trial Register NTR5037. Registered 15 April 2015). The trial has approval of the faculty Ethics Committee of the Radboud University (number: ECSW2015-0903-303).

## Participants

### Recruitment

‘Take it personal!’ was developed for adolescents (14–21 years) with mild to borderline ID who are admitted to treatment centers in the Netherlands. These adolescents often have severe internalizing (anxiety, depression) and externalizing (aggression, antisocial behavior) behavioral problems or psychiatric diagnoses. Participants will be recruited through treatment centers. About ten treatment centers in the Netherlands will be invited to participate in this RCT. These treatment centers offer specialized residential and extramural care for adolescents with mild to borderline ID and severe behavioral problems. A team of therapists will implement the intervention and behavioral scientists and participating institutions will receive the materials for ‘Take it personal!’ free of charge.

After selecting the treatment centers, adolescents will complete a screening questionnaire to identify adolescents who meet the inclusionary criteria. Adolescents will be eligible to enter the trial if they meet the following inclusion criteria: (1) life time prevalence of alcohol, cannabis, or hard drug use, (2) belonging to one of the four personality high-risk groups (SS, IMP, AS or NT), and (3) providing signed informed consent along with the signed informed consent from parents or legal representative. Adolescents scoring more than one standard deviation above the sample mean on one of the four high-risk personality scales of the Substance Use Risk Profile Scale (SURPS) [40] will be classified as belonging to that risk profile. Adolescents who will score above the average on more than one personality profile will be assigned to the profile for which they showed the largest statistical deviation with respect to the z-score (cf. [17]). Adolescents with addiction problems will be excluded from participation in the intervention and the trial because this intervention is not considered sufficient for them. For these adolescents, treatment will be organized in collaboration with a regional institute for addiction care. Parents or legal representatives will be informed about the study through a letter sent home, asking them to contact the researchers by phone or email if they do not wish their child to participate in the screening (passive informed consent). Adolescents will be informed on the day of the screening, at which time they have the option not to participate in the trial. Parents (when participants are under the age of 18 years) and adolescents will need to provide active informed consent to participate in the RCT. After the screening questionnaire and informed consent, the adolescents will be individually randomized to either control condition or experimental condition.

### Power analyses

In the present study, we aimed to show a medium effect size (cf. [14]). Power-analysis was conducted based on an average effect size of f = 0.25 [41], a 2-sided test at alpha = .05, a statistical power (1-beta) of 0.80, and 10 % loss-to-follow-up after randomization. Based on these assumptions a sample size of 140 adolescents (70 in each condition; G-power) is required. Eighteen intervention groups are required, assuming that up to a maximum of four participants will be included in intervention group.

### Intervention

Adolescents in the intervention group will receive the intervention ‘Take it personal!’ and care as usual. Adolescents will participate in one of the four versions of the intervention that address each high-risk personality trait. ‘Take it personal!’ comprises three main components: (1) psycho-education, (2) behavioral coping skills, and (3) cognitive coping skills. The first phase the intervention focuses on psycho-education regarding the participants’ personality profile and coherent problematic coping behavior, like substance abuse or aggression. In this phase, participants are motivated to become familiar with their personality profile and learn to deal with their personality through exercises. Daily life experiences and coherent physical, cognitive, and behavioral reactions will be analyzed. Participants will set individual goals, which they will try to achieve during the training. The coping skills training will engage adolescents in activities aimed at recognizing automatic thoughts. Participants will identify personality-specific thoughts that lead to problematic behavior. For example, the intervention aimed at adolescents with the personality profile ‘Impulsive’ will focus on thinking before taking action. Simultaneously, the participants will be trained to use cognitive restructuring techniques to counter such thoughts. Participants will edit a personalized ‘changing plan’ to deal differently with their problematic and risky behavior.

The intervention will involve five group sessions and six individual sessions spread across six weeks. One individual and one group session will be conducted every week, except for week 5 during which only one individual session will be offered. This week will be used to give participants extra time to practice the assigned tasks in their daily lives. Individual sessions will last 30 minutes and group sessions will last 45 minutes. During individual sessions, the trainer and participant will prepare that week’s group session. During the individual sessions, the participants will be able to pick and bring a confidant from their team of supervisors. This will increase the generalization to everyday life and ensures that participants feel prepared and secure during the group sessions.

Two qualified trainers, one psychomotor therapist and a behavioral scientist, will carry out the intervention. A psychomotor therapist will have to be present because of the exercises based on psychomotor principles while a behavioral scientist needs to be involved because of the principles of CBT and MI. Training should be provided by a behavioral scientist who is experienced with these techniques. All trainers will participate in a two-day training on the principles of CBT and MI and all sessions will be practiced. All group sessions will be carried out in the psychomotor department of the home institution. Individual sessions will be held in flexible rooms.

Each individual and group session will have the same structure. Each individual session will start by asking the participant’s confidant what he or she has learned in the last group session and what exercises they have done. Subsequently, the trainer and participant will do some exercises and assignments. In the individual session, participants will be asked to give examples from their daily lives. These examples will be used to complete exercises within the group sessions. Participants’ confidants will play a supporting role in the individual session. Afterward, the participant and trainer will prepare the next group session. All group sessions will start by offering participants some refreshments to make them feel at ease and secure. These sessions will comprise exercises, games, and psychomotor practices prepared during the individual sessions. Every group session will comprise at least one exercise from psychomotor therapy. To close the group sessions, the trainers and participants will summarize that group session together. The training will be developed according to the principles of CBT, and it will be adjusted to the cognitive capacities of adolescents with mild to borderline ID.

Adolescents assigned to the control group will receive no further intervention, but they will receive ‘care as usual’. ‘Care as usual’ will not be standardized or protocolled, but we will make an inventory of other prevention and intervention programs aimed at substance use. Most adolescents receive treatment for their own specific problems; some of them receive residential care while others receive extramural treatment. In both cases, treatment is formulated through personal goals in a so called ‘individual treatment plan’. A multidisciplinary team is involved in the treatment of each adolescent. All participating adolescents will be rewarded with a small gift.

## Data collection

During the pre-test, the participants will complete the SURPS-NL-LVG questionnaire (SURPS-NL-LVG; [40]; custom version SURPS; [42]). Based on their scores on this questionnaire, participants will be classified into one out of the four personality profiles (SS, IMP, AS, and NT).

### Outcomes

‘Take it Personal!’ aims to decrease substance use among adolescents with mild and borderline ID. We operationalized goals for limiting alcohol, cannabis, and hard drug use. The primary outcome of alcohol use will be the percentage of decrease in binge drinking, weekly use, and problematic use. The primary outcomes of cannabis use will be the percentage of decrease in lifetime cannabis use and weekly use. The primary outcome of hard drug use will be the percentage of decrease in lifetime use. These outcome measures will be assessed at baseline and two, six, and twelfth months after the intervention using the Substance Use and Misuse among Intellectually Disabled Persons Questionnaire (SumID-Q; [43]). This questionnaire is specifically developed to measure substance use among people with intellectual disabilities. Secondary outcomes are the intention to use less alcohol and/or drugs in the future [44, 31], motives for alcohol and/or drug use (Drinking Motives Questionnaire-Revised-Short Form (DMQ-R-SF) [45], and internalizing and externalizing behavioural problems measured by YSR [46]. Adolescents with mild to borderline ID are able to complete self-report instruments with some support [47]. Therefore, all measures will be supported using structured interviews.

This study examined the effectiveness of ‘Take it Personal!’ in daily practice (effectiveness trial). In this case, it is important to monitor the program fidelity [48, 49]. For this reason, we will monitor five domains: 1) adherence, 2) exposure (dosage), 3) quality of the delivery, 4) responsiveness of the participant, and 5) program differentiation (cf. [49]).

### Statistical analysis

Descriptive analyses will be conducted to examine whether the randomization results in a similar distribution of demographic factors and outcome measures in both conditions. Variables that show different distributions between the two groups will be entered as confounders in all models testing the effectiveness of the intervention. The effect of the intervention program on the primary and secondary outcome variables will be tested in accordance with the intention-to-treat principle and in a completers-only framework by using Mplus [50]. Intention-to-treat means that all participants will be analyzed in the condition to which they will be assigned by randomization. Missing data will be handled by multiple imputation (MI). A total of 20 datasets will be completed by multiple imputation. Mplus will read the 20 datasets via the TYPE = IMPUTATION option and will carry out the desired analyses for each dataset. Mediating the parameter estimates will then aggregate the results for the 20 analyses. With respect to the completers-only analyses, only the participants with scores for all time points will be included. In both the intention-to-treat and the completers-only analyses, the effect of the intervention condition will be compared to the control condition. Because the data have a multilevel structure (i.e., individuals are ‘clustered’ within treatment centres), the individual respondents within treatment centres may be interdependent. To correct for the potential non-independence (complexity) of the data, the TYPE = COMPLEX procedure in Mplus will be used. This procedure corrects the standard errors of the parameter estimates for dependency, leading to unbiased estimates. The results of the study will be reported in accordance with the CONSORT statement [51, 52].

## Discussion

The present paper described the study protocol to test the effectiveness of the secondary preventive program called ‘Take it personal!’ by means of a Randomized Controlled Trial. The intervention aims to prevent adolescents with mild to borderline ID from problematic substance use by helping them develop competences to deal with their personality traits. It is hypothesized that adolescents in the intervention group will show a higher reduction in substance use compared to adolescents in the control group at follow-up.

### Strengths and limitations

The intervention has several strengths. First, this intervention is the first secondary prevention program in the Netherlands for adolescents with mild to borderline ID that focuses on helping adolescents acquire skills to deal with high-risk personality profile. Second, the program is based on a proven effective intervention [14–17]. Third, the program incorporates elements of CBT and MI techniques, which have been proven effective for adolescents with mild to borderline ID [35–37]. Fourth, ‘Take it personal!’ will fill an important gap with regard to prevention programs for adolescents with mild to borderline ID. Fifth, the intervention has been developed especially for the target group according to proven effective techniques, like psychomotor therapy and the guidelines for effective interventions for people with mild ID [39]. This is an important strength, as research has shown that interventions for non-disabled peers are insufficient for people with mild to borderline ID [39]. Six, during the individual sessions, the participants will be able to bring their own confidants from their supervising team, which will increase the generalization to everyday life and ensure that participants will feel prepared and secure during the group sessions. The importance of a RCT is underlined by the fact that nationally and internationally, no scientific evidence supports the effectiveness of current prevention programs on adolescents with mild to borderline ID [10, 12].

A limitation of this study is that the behavior data as well as placement in intervention groups (SS, IMP, AS, NT) will be based on self-reports, which might lead to measurement errors. However, research has shown that adolescents with mild and borderline ID are able to complete self-report instruments, although with some support [47]. Accordingly, all measurements will be done under the supervision by means of structured interviews.

### Implications for practice

If the intervention proves to be effective in preventing substance use by adolescents with mild to borderline ID, the study will have strong practical relevance for secondary prevention and intervention programs. It could reduce healthcare costs to society, as adolescents with addiction disorder provide major social risks and costs (for example, costs associated with addiction care as well as social problems like theft, vandalism, aggression etc.).

## Conclusion

This paper describes an effectiveness study design of a secondary preventive program developed for substance-using adolescents with mild to borderline ID. Evaluation of the intervention will provide insights into the effectiveness of ‘Take it personal!’ prevention program.
